# Analysis of Factors Associated With Variability and Acidosis of the Umbilical Artery pH at Birth

**DOI:** 10.3389/fped.2021.650555

**Published:** 2021-05-25

**Authors:** María Luisa Mayol Pérez, José Manuel Hernández Garre, Paloma Echevarría Pérez

**Affiliations:** ^1^Department of Health Sciences Program, Universidad Católica de Murcia (UCAM), Guadalupe, Murcia, Spain; ^2^Hospital Torrevieja, Torrevieja, Spain; ^3^Department of Political Sciences, Social Anthropology and Public Finance University of Murcia, Murcia, Spain; ^4^Hospital Rafael Méndez Lorca, Lorca, Spain

**Keywords:** birth asphyxia, umbilical cord blood, fetal hypoxia, umbilical artery, pH

## Abstract

**Background:** Perinatal asphyxia is a significant contributing factor for neonatal morbidity and mortality. The aim of this study was to investigate the clinical factors associated with umbilical artery pH variability and fetal acidosis at birth.

**Methods:** This is a single center cross-sectional study in a public regional hospital in southeastern Spain from January to December 2019. The reference population was 1.655 newborns, final sample of 312 experimental units with validated values of umbilical cord blood pH.

**Results:** Factors such as gestational age at term (${\bar{X}}_{\text{at}-\text{term}}$: 7.26 ± 0.08-${\bar{X}}_{\text{no}}$-_at−term_: 7.31 ± 0.05, *p*: 0.00), primiparity (${\bar{X}}_{\text{primiparity}}$: 7.24 ± 0.078-${\bar{X}}_{\text{multiparity}}$: 7.27 ± 0.08, *p*: 0.01), induced labor (${\bar{X}}_{\text{induced}}$: 7.24 ± 0.07-${\bar{X}}_{\text{spontaneous}}$: 7.26 ± 0.081, *p*: 0.02), vaginal delivery (${\bar{X}}_{\text{vaginal}}$:7.25 ± 0.08-${\bar{X}}_{\text{cesarean}}$:7.27 ± 0.07, *p*: 0.01), and prolonged dilation duration (${\bar{X}}_{\text{AboveAverage}}$: 7.22 ± 0.07-${\bar{X}}_{\text{BelowAverage}}$: 7.27 ± 0.08, *p*: 0.00), expulsion duration (${\bar{X}}_{\text{AboveAverage}}$: 7.23 ± 0.07-${\bar{X}}_{\text{BelowAverage}}$: 7.26 ± 0.08, *p*: 0.01), and total labor duration (${\bar{X}}_{\text{AboveAverage}}$: 7.23 ± 0.07-${\bar{X}}_{\text{BelowAverage}}$: 7.27 ± 0.08, *p*: 0.00) are associated with a decrease in umbilical artery pH at birth. However, only three factors are associated with acidosis pH (<7.20) of the umbilical artery at birth: the induction of labor [OR: 1.74 (95% CI: 0.98–3.10); *p*: 0.04], vaginal delivery [OR: 2.09 (95% CI: 0.95–4.61); *p*: 0.04], and total duration of labor [OR: 2.06 (95% CI: 1.18–3.57); *p*: 0.01].

**Conclusions:** Although several factors may affect the variability of umbilical artery pH at birth by decreasing their mean values (gestational age, primiparity, induced labor, vaginal delivery and prolonged: dilation duration, expulsion duration and total labor duration), only induction of labor, vaginal delivery and total duration of labor are associated with an acidosis (<7.20) of same.

## Introduction

The term asphyxia can be defined as an alteration in gas exchange that leads to progressive hypoxia, hypercapnia, and acidosis (1). In the case of neonatal asphyxia, most of its complications are transient, but in the case of prolonged exposure, the central nervous system can be affected, becoming one of the main causes of mortality and neurological complications (2).

Mortality and morbidity associated with intrapartum hypoxia persists as a global health problem (3), being one of the main causes of neonatal mortality in the first 24 h (4). According to the World Health Organization (WHO) statistics, 3% of children (3.6 million children) suffer from moderate to severe intrapartum asphyxia (4). Of these, 23% die, and around 20% suffer from associated sequelae that vary according to prognosis, severity of symptoms, risk factors, and patient management (2, 5).

Neonatal asphyxia alters the passage from intrauterine to extrauterine life, a transit that requires well-orchestrated measures to ensure neonatal survival (6). In this sense, the analysis of the acid–base balance of the umbilical artery blood at birth is an objective way to evaluate the metabolic state of the newborn, noting the presence or absence of neonatal asphyxia (7), a measure that is internationally accepted as a criterion for defining intrapartum hypoxia (8). The analysis of the variables associated with the variability and acidosis of umbilical artery pH at birth is, therefore, the best way to approach the comprehension of factors associated with the development of fetal asphyxia. Identification of the risk factors leads us to more effective interventions against the principal causes and, accordingly, affords us better knowledge not only of the causes associated with fetal acidosis but also of the contributing factors to worse average values of the principal fetal gasometry measures at birth. Our discussion is not solely from a pathology perspective, as we are dealing with a range of high-risk situations constituting a scenario where it is more likely for a complication to develop; this study, thus, advances our understanding of fetal acidosis and consequently contributes toward facilitating the clinical management of this process. The aim of this study was to investigate the clinical factors associated with umbilical artery pH variability and fetal acidosis at birth.

## Materials and Methods

### Setting and Participants

This is a cross-sectional study. The reference population was the 1.655 newborn during the year 2019 between January and December in a hospital, in the southeast of Spain (Hospital Rafael Méndez Lorca), belonging to the National Health System. The Hospital Rafael Méndez Lorca is a regional public health hospital within the Servicio Murciano de Salud (Murcia Health Service) hospital network; it provides health care to residents of Murcia Health Area III (Spain), numbering 172.630 inhabitants in total. The hospital has its own Maternal and Child Service, with a specialized Obstetrics–Gynecology Department.

The sample population comprises the births that occurred in the reference hospital and met the following criteria: hospital births during the study period for which gasometry data and documentation of umbilical cord blood samples, both arterial and venous, were recorded; home births were not included due to the absence of arterial and venous cord blood gasometry records. Exclusion criteria: fetuses that died *in utero*; births where the umbilical gas analysis was performed after 15 min, since in such cases, fetal acidosis is less precise as a diagnostic method (9); and hospital births lacking complete umbilical cord gasometry data (it was necessary to have both arterial and venous cord blood samples). We excluded results from extracted samples that showed a probability of having been mixed or taken from the same blood vessel, as these were accordingly considered invalid (9, 10) (arterial pH is lower than venous pH, and when the pH difference between the two samples is <0.02 and the difference in carbon dioxide partial pressure is <5 mm Hg or 0.7 kPa (kilopascals), this would indicate possible contamination from the umbilical vein or the air, it being almost impossible for a pCO_2_ of <22 mm Hg to occur in the umbilical artery).

For the selection of the sample, a simple random selection process was performed after excluding 26 infants: five due to intrauterine death and 21 for not having a registry of umbilical artery blood gases at birth. The final sample size was 312 infants, assuming a precision of 5.0% in estimating a proportion by using a normal asymptotic confidence interval with a correction for finite populations at 95% bilaterally, and taking the worst unfavorable proportion of 50%, although we know from previous studies (1, 4) that intrapartum acute fetal compromise occurred in a much lower proportion, that is, between 2 and 3%. Sample collection was done based on the simple random sampling technique applied to the study population of 1.655 women, all with their corresponding sequential number in accordance with the date of the birth. The random number table was obtained using the MAS 2.1 program produced by the Glaxo W. laboratory.

### Study Data

The data were obtained through the computerized clinical data record (SELENE) of the reference hospital. SELENE, the clinical station of the Murcia Health System's Corporate Hospital Information System, affords multiple ways to access a patient's clinical history. Each clinical history is divided into healthcare processes, each of which contains information in the form of clinical records (forms, notes, reports, and requests).

In order to analyze the collected information, the data provided through the Hospital database (SELENE) was converted and coded into an Excel spreadsheet, in order to facilitate its subsequent loading into the SPSS 26.0 program for data analysis.

The measurement technique and instrumentation employed was the biochemical analysis of umbilical cord blood (arterial and venous) via the GEM Premier 4000 approved pH meter (Werfen, Le Pré-Saint-Gervais, France).

The umbilical cord blood collection technique involves placing one clamp close to the newborn's navel and a second at the end of the umbilical cord, without interfering with the recommended umbilical cord clamping delay of at least 30–60 s post-birth (11) and without prolonging the clamping for 120 s, as recent studies have shown [the observational studies showed delayed cord clamping up to 120 s had effects on both arterial and venous cord blood gas values, the magnitude of this effect is clinically insignificant in healthy, term, vaginally delivered newborns (12)]. In our study the cord was clamped at 60 s.

The blood is extracted, with the least amount of air possible, into two separate 1- or 2-ml syringes pretreated with heparin. After the blood has been extracted, any air bubbles present in the syringes must be eliminated, and the blood gas analysis is performed in a calibrated apparatus, in this case, the GEM Premier 4000 approved pH meter. The analysis must be performed within the first 15 min.

### Outcome Measures

The main outcome measures were the variability and acidosis of the umbilical artery pH at birth, identifying as such the arterial pH range <7.20 (13, 14). According to the World Health Organization (WHO), perinatal asphyxia is characterized by pH <7.20 on umbilical cord arterial blood sample (15).

The value of fetal pH at birth is obtained by analyzing the acid–base equilibrium of umbilical cord blood and is considered the best technique for establishing a diagnosis of fetal asphyxia (9). For our study, we used a cutoff value of <7.20 as per the World Health Organization (15); in line with acidosis type classification, umbilical arterial blood gasometry values of <7.20 were considered to be acidotic. Classifications by composition were as follows: respiratory acidosis [pH <7.20, carbon dioxide partial pressure (pCO_2_) > 60 mmHg, base excess within normal limits (more than −12 mEq/L)], and mixed acidosis [pH <7.20, carbon dioxide partial pressure (pCO_2_) >60 mmHg, base excess less than or equal to −12 mEq/l] (10).

### Independent Variables

Dependent variables that were crossed with different independent variables such as:

Maternal age (not advanced maternal age <35 years old/advanced maternal age >35 years old) and nationality (Spanish/foreign).

Gestational age (normal: at term 37–42 weeks/abnormal: preterm <37 weeks and >42 weeks post-term), parity (primiparity/multiparity).

Type of onset of labor (spontaneous: commenced naturally due to normal physiological mechanisms without any type of external intervention occurring/induced: commenced artificially *via* the use of mechanical or pharmacological means that produce contractions). According to reasons for induction: retarded intrauterine growth (IUGR): growth percentile <3/growth percentile between 3 and 10 with Doppler hemodynamic alteration; risk of loss of fetal well-being (RLFW): pathological or non-reactive cardiotocographic trace that after performing a biophysical profile <8/10, or a persistently non-reactive cardiotocographic trace at 12–24 h despite having obtained a biophysical profile of 8/10; prolonged pregnancy (PP):(>42 weeks gestation); premature rupture of membranes (PROM): premature rupture of amniotic membranes before birth onset; oligohydramnios: maximum amniotic fluid column <2 cm; maternal disease unrelated to the pregnancy: maternal disease indicating end of gestation and pregnancy-induced hypertension (PIH): hypertension that appears after 20 weeks of gestation, subdivided into gestational hypertension and pre-eclampsia.

Type of delivery: vaginal [eutocic: vaginal birth without any obstetric instrument intervention; instrumental: vaginal birth via the use of various instruments (suction cup, spatula or forceps)/cesarean (elective or scheduled cesarean: gestating women with maternal or fetal pathologies contraindicating or counseling against vaginal birth; intrapartum cesarean: once labor has commenced)].

Type of amniorexis [spontaneous: rupture of the fetal membranes without external action/artificial: rupture of the fetal membranes effected by the health professional through performance of an amniotomy (artificial perforation of the sac)], presence or absence of episiotomy (no episiotomy/yes episiotomy), sex of the newborn (female/male), presence or absence of nuchal cord at birth (no presence/yes presence).

Presence or absence of analgesia (without analgesia: no type of analgesia/with analgesia: epidural, spinal or general).

Neonatal weight at birth [normal (≥2,500 g and <4,000 g) and abnormal (underweight <2,500 g and macrosomia ≥4,000 g)].

Time elapsed since the rupture of the amniotic sac [below the study sample average (<443 min)/above the study sample average (>443 min)].

Duration of the dilation stage [below the study sample average (<261 min)/above the study sample average (>261 min)].

Duration of the expulsion stage [below the study sample average (<44 min)/above the study sample average (>44 min)].

Duration of delivery [below the study sample average (<312 min)/above the study sample average (>312 min)].

### Statistical Analysis

First, a descriptive analysis was performed with the objective of calculating the proportion of fetal acidosis in the sample. After that, and with the objective of analyzing the factors associated with the decrease in umbilical artery pH at birth, a bivariate analysis was performed with the dependent variable ungrouped, applying a *t*-student test in the cases where the independent variables were dichotomous and an analysis of variance (one-way ANOVA) when they had more than two categories. Subsequently, in order to distinguish the factors associated with fetal acidosis at birth, a bivariate analysis was performed with the dependent variable grouped into two categories (normal pH >7.20, acidic pH <7.20), applying Pearson's Chi-square test and the Yates correction when the observed frequencies were <5. Finally, in order to analyze the magnitude of the adjusted association of the dependent variable with the independent variables, the variables for colinearity were tested before entering them in the model. Gestational age, parity, labor onset, type of delivery or prolonged durations of dilatation, expulsion, and total labor are intrapartum variables that can be determinant in the variations of blood gases at birth (15–17) and had presented a prior significant association in the bivariate analysis.

A multivariate logistic regression analysis was performed using the enter method to obtain the regression models, introducing into the matrix only those variables that had presented a prior significant association in the bivariate analysis and after eliminating the variables that presented collinearity (dilation time and delivery time were colinear with the total duration of labor, being fractions of it). In the different hypothesis tests, the results obtained for a value of *p* <0.05 were accepted as significant.

## Results

Of the 312 infants constituting the sample, 30.7% (96) presented acidotic pH of the umbilical artery at birth, 79.2% (76) showed respiratory acidosis, and 20.8% (20) showed mixed acidosis (Table 1).

**Table 1:** Frequencies of neonates who developed acidosis.

	**Frequency**	**Percentage**	**Accumulated percentage**
Neonates without acidosis (pH >7.20)	216	69.2	79.2
Neonates with acidosis (pH <7.20)	96	30.7	100.0
Total	312	100.0	
**Neonates with acidosis**
Respiratory acidosis	76	79.2	79.2
Mixed acidosis	20	20.8	100.0
Total	96	100.0	

### Analysis of the Variability of Umbilical Artery pH at Birth

There is a statistically significant association between the decrease in umbilical artery pH mean values and variables such as gestational age at term, primiparity, induced labor, vaginal delivery, and the duration of dilation, duration of expulsion, and total duration of labor above average. No significant association is observed with other variables such as maternal age, maternal nationality, amniotic sac rupture type, presence or absence of episiotomy, presence or absence of analgesia, sex of the newborn, neonate weight, presence of nuchal cords, or above-average duration of rupture of the amniotic sac (Table 2).

**Table 2:** Mean values for the umbilical artery pH as a function of clinical variables.

**Variables**	***N***	**x**	**S**	***P***
**Maternal age**				
Not advanced <35 years old	225	7.25	0.08	0.11
Advanced >35 years old	87	7.27	0.08	
**Nationality**				
Spanish	205	7.26	0.08	0.78
Foreign	107	7.27	0.07	
**Gestational age**				
Normal (37–42 weeks)	296	7.26	0.08	0.00
Abnormal (<37 or >42 weeks)	16	7.31	0.05	
**Parity**				
Primiparity	131	7.24	0.08	0.01
Multiparity	181	7.27	0.08	
**Labor onset**				
Spontaneous	177	7.26	0.08	0.02
Induced	97	7.24	0.07	
**Type of delivery**				
Cesarean	86	7.27	0.07	0.01
Vaginal	226	7.25	0.08	
**Amniotic sac rupture type**				
Spontaneous	153	7.25	0.08	0.45
Artificial	152	7.26	0.07	
**Analgesia**				
Without analgesia	127	7.26	0.08	0.32
With analgesia	183	7.25	0.08	
**Episiotomy**				
No episiotomy	150	7.25	0.08	0.93
Yes episiotomy	102	7.25	0.08	
**Newborn's sex**				
Female	154	7.26	0.08	0.79
Male	158	7.27	0.08	
**Birth weight**				
Normal (≥2,500 g and <4,000 g)	279	7.26	0.08	0.69
Abnormal(<2,500 g and ≥4,000 g)	33	7.25	0.08	
**Nuchal cord**				
No	241	7.26	0.08	0.76
Yes	67	7.25	0.05	
**Amniotic sac rupture period**				
Below average (<443 min)	184	7.26	0.08	0.13
Above average(≥443 min)	90	7.24	0.08	
**Dilation duration**				
Below average (<261 min)	165	7.27	0.08	0.00
Above average(≥261 min)	103	7.22	0.07	
**Expulsion duration**				
Below average (<44 min)	191	7.26	0.08	0.01
Above average (≥44 min)	80	7.23	0.07	
**Total duration of labor**				
Below average (<312 min)	159	7.27	0.08	0.00
Above average(≥312 min)	119	7.23	0.07	

*G, grams; n, number; x, mean; s, standard deviation; p, p-value*.

The results do not show significant differences according to reasons for induction [*F*_(6)_: 0.74, *p*: 0.61], despite the fact that the lowest mean values of umbilical artery pH are found in births whose induction was retarded intrauterine growth (IUGR) ($\bar{X}$: 7.20 ± 0.06), followed by a risk of loss of fetal well-being (RLFW) ($\bar{X}$: 7.22 ± 0.06), prolonged pregnancy (PP) ($\bar{X}$: 7.23 ± 0.07), premature rupture of membranes (PROM) ($\bar{X}$: 7.24 ± 0.08), oligohydramnios ($\bar{X}$: 7.24 ± 0.07), maternal disease unrelated to the pregnancy ($\bar{X}$: 7.26 ± 0.07), and pregnancy-induced hypertension (PIH) ($\bar{X}$: 7.29 ± 0.06). Regarding the type of completion of delivery, a significant association was observed with respect to the decrease in the mean values of the umbilical artery pH in instrumented delivery compared with elective cesarean section (${\bar{X}}_{\text{instrumented}}$: 7.24 ± 0.07$\sim {\bar{X}}_{\text{cesarean}}$: 7.29 ± 0.06; *t*_(100)_:−3.92, *p*:0.00), an association that is not found in other types of delivery such as eutocic delivery(${\bar{X}}_{\text{instrumented}}$: 7.24 ± 0.07$\sim {\bar{X}}_{\text{eutocic}-}$: 7.26 ± 0.08; *t*_(223)_: −1.56, *p*:0.12) or intrapartum cesarean section (${\bar{X}}_{\text{instrumented}}$: 7.24 ± 0.07$\sim {\bar{X}}_{\text{intrapartum}-\text{cesarean}}$: 7.26 ± 0.08; *t*_(106)_: −1.64, *p:*0.10), even though an instrumented delivery has lower mean values of umbilical artery pH than these.

### Analysis of Acidosis of Umbilical Artery pH at Birth

The bivariate analysis with the clustered dependent variable shows a statistically significant association between the presence of umbilical artery pH at birth and factors such as gestational age at term [OR: 6.37 (95% CI: 0.85–47.52)], primiparity [OR: 1.32 (95% CI: 1.04–1.68)], onset of induced labor [OR: 1.76 (95% CI: 1.04–2.96)],vaginal delivery [OR: 2.06 (95% CI: 1.13–3.74)] and duration of dilation [OR: 2.63 (95% CI: 1.55–4.44)], duration of expulsion [OR: 1.74 (95% CI: 1.00–2.99)], and total duration of labor [OR: 2.34 (95% CI: 1.43–3.99)] above average (Table 3).

**Table 3:** Bivariate analysis of umbilical artery pH acidosis as a function of clinical variables.

**Variables**	**Fetal pH**		**Results**	
	**Normal ≥ 7.20 (*n*: 219)**	**Pathological <7.20 (*n*: 93)**	**X^**2**^**	**OR**
			***P***	**95% CI**
Not advanced age (<35 years old)	151 (67.1%)	74 (32.9%)	3.66	0.57 (0.32–1.02)
Advanced age (>35 years old)	68 (78.2%)	19 (21.8%)	0.06	
Spanish nationality	142 (69.3%)	63 (30.7%)	0.24	0.79 (0.52–1.47)
Foreign nationality	77 (72%)	30 (28%)	0.62	
Normal gestational age (37–42 weeks)	204 (68.9%)	92 (31.1%)	4.47	6.37 (0.85–47.52)
Abnormal gestational age (<37 or >42 weeks)	15 (93.7%)	1 (6.3%)	0.03	
Primiparity	82 (62.6%)	49 (37.4%)	6.23	1.32 (1.04–1.68)
Multiparity	137 (75.7%)	44 (24.3%)	0.01	
Spontaneous	128 (72.3%)	49 (27.7%)	4.50	1.76 (1.04–2.96)
Induced	58 (59.8%)	39 (40.2%)	0.03	
Cesarean birth	69 (80.2%)	17 (19.8%)	5.72	2.06 (1.13–3.74)
Vaginal delivery	150 (66.4%)	76 (33.6%)	0.02	
Spontaneous amniorexis	104 (67.9%)	49 (32.1%)	0.51	0.84 (0.51–1.37)
Artificial amniorexis	109 (71.7%)	43 (28.3%)	0.48	
Without analgesia	92 (72.4%)	35 (27.6%)	0.61	1.22 (0.74–2.00)
With analgesia	125 (68.3%)	58 (31.7%)	0.44	
No episiotomy	98 (65.3%)	52 (34.7%)	0.50	0.82 (0.48–1.41)
Episiotomy	71 (69.6%)	31 (30.4%)	0.48	
Female sex	111 (72.1%)	43 (27.9%)	0.52	1.19 (0.74–1.94)
Male sex	108 (68.4%)	50 (31.6%)	0.47	
Normal weight of newborn (≥2,500 g and <4,000 g)	200 (71.7%)	79 (28.3%)	2.81	1.86 (0.89–3.09)
Abnormal weight of newborn (<2,500 g and ≥ 4,000 g)	19 (57.6%)	14 (42.4%)	0.09	
No nuchal cord	169 (70.1%)	72 (29.9%)	0.06	0.93 (0.51–1.69)
Nuchal cord	48 (71.6%)	19 (28.3%)	0.81	
Amniotic sac rupture period below average(<443 min)	126 (69.6%)	55 (30.4%)	1.08	1.32 (0.78–2.26)
Amniotic sac rupture period above average (>443 min)	57 (63.3%)	33 (36.7%)	0.29	
Dilation duration below average (<261 min)	125 (75.7%)	40 (24.2%)	13.23	2.63 (1.55–4.44)
Dilation duration above average (>261 min)	56 (54.4%)	47 (45.6%)	0.00	
Expulsion duration below average (<44 min)	136 (71.2%)	55 (28.8%)	3.988	1.74 (1.00 2.99)
Expulsion duration above average (>44 min)	47 (58.8%)	33 (41.3%)	0.04	
Total duration of labor Below average (<312 min)	121 (76.10%)	38 (23.9%)	11.239	2.34 (1.43–3.99)
Total duration of labor above average (>312 min)	68 (57.1%)	51 (42.9%)	0.00	

*X^2^, chi-squared; p, p-value, OR, odds ratio; 95% CI, confidence interval (95%)*.

No significant association was observed with other variables such as maternal age, maternal nationality, amniotic sac rupture type, presence or absence of episiotomy, presence or absence of analgesia, sex of the newborn, neonate weight, presence of nuchal cords, or above-average duration of rupture of the amniotic sac (Table 3).

However, once we introduced into the same matrix those variables that were significant in the bivariate analysis and after performing the collinearity analysis, in order to analyze the adjusted association of the independent variables with the response variable, a colinearity was found between the variables duration of dilation, duration of expulsion, and total duration of labor (eigenvalue 0.009 with condition index 74.171 and variance ratios of 0.98 for total duration, 0.97 for dilation time, and 0.59 for duration of expulsion). After eliminating from the matrix dilation time and duration of expulsión (fractions of total duration of labor), it is observed that there is no colinearity between the variables (eigenvalue of 2.728 with condition index 1 and proportions of variance of 0.00 for gestational age and 0.04 for total duration of labor), with a variance inflation factor (VIF) <10 (1), condition index <30 (3.16), and proportion of variance <0.5.

We observed how the multivariate analysis of logistic regression shows a significant association between the presence of acidosis of umbilical artery pH at birth and the onset of induced labor [OR: 1.74 (95% CI: 0.98–3.10); *p*: 0.04], completion of vaginal delivery [OR: 2.09 (95% CI: 0.95–4.61); *p*:0.04] and total duration of labor [OR: 2.06 (95% CI: 1.18–3.57); *p*:0.01] (Table 4).

**Table 4:** Multivariate analysis of umbilical artery pH acidosis as a function of clinical variables.

**Variables**	**β**	***P***	**OR**	**95% CI**
**Gestational age**				
Normal (37–42 weeks)	Reference	0.06	0.13	(0.01–1.12)
Abnormal (<37–>42 weeks)	−1.98			
**Parity**				
Primiparity	Reference	0.23	0.71	(0.40–1.24)
Multiparity	−0.33			
**Labor onset**				
Spontaneous	Reference	0.04	1.74	(0.98–3.10)
Induced	0.55			
**Type of delivery**				
Cesarean	Reference	0.04	2.09	(0.95–4.61)
Vaginal	0.74			
**Total duration of labor**				
Below average (<312 min)	Reference	0.01	2.06	(1.18–3.57)
Above average(≥312 min)	0.72			

*β, beta error; p, p-value; OR, odds ratio; 95%CI, confidence interval (95%)*.

## Discussion

### Main Findings

Factors such as gestational age, primiparity, labor induction, vaginal delivery, or prolonged durations of dilatation, expulsion, and total labor decrease the umbilical artery pH at birth, but only induction of labor, vaginal delivery, and total duration of labor are associated with an acidosis of the same. The inductions of retarded intrauterine growth and instrumented delivery have the lowest mean umbilical artery pH at birth according to induction reason and type of labor completion.

### Interpretation

Our research reflects worse average values in the biochemical parameters of newborns at birth, and a higher percentage of acidosis in the normal gestational age range (37 to 41 gestational weeks inclusive) compared with the abnormal gestational age range (<37 or >41 weeks). This aligns with authors such as Parikh et al. (18) and Sandström et al. (19), with an increased rate of admission to intensive care units and higher respiratory morbidity in cases lying closer to gestational week 37, Eskes et al. (20), Little et al. (21), and Yi et al. (17) reaffirm that birth at 37 gestational weeks is associated with a higher frequency of clinically relevant adverse perinatal outcomes. Term babies are more susceptible to mechanical injury and progressive hypoxic insults associated with prolonged and obstructed labor, which may often warrant operative intervention (17).

Regarding parity, it has been shown that perinatal asphyxia is more common in newborns of primiparous mothers (22, 23). In our research, we observed an association between primiparity and decreased mean umbilical artery pH. Tawse et al. (24) report that in the case of primiparous mothers, the risk of at-birth asphyxia is three times higher than for multiparous mothers {ORa: 3.10 [95% IC (1.51–6.38)]}. Their results are similar to those obtained by Igboanugo et al. (25), who observed that first-time mothers showed an increased odds ratio for at-birth asphyxia (OR: 2.64). Ilah et al. (26), Ensing et al. (22), Crovetto et al. (16), or Liljestrom et al. (27) confirm a higher incidence of adverse perinatal outcomes, fetal asphyxia, and neonatal hypoxia in primiparous women. While nulliparity is a risk factor for adverse neonatal outcomes (28) this risk may be influenced by the increased incidence of delay in the second stage of labor and of operative birth, particularly instrumental and emergency cesarean delivery, which significantly increase the risk of poor outcomes (17).

Induction of labor has also been related to intrapartum fetal acidosis (29–32). Gudayu et al. (33) reported a significant relationship (*p* <0.001) between induced birth and low 5-min Apgar test scores in comparison with spontaneous-onset birth. Yi et al. (17) found serious neonatal outcomes in induced births [ORa: 1.08 (95% IC: 1.01–1.15); *p*:0.03], and Whittington et al. (34) conclude that even with successful vaginal delivery of a medically indicated induction of labor, the risk for adverse outcomes remains elevated (neonates with Apgar scores <7 at 5 min and acidotic values in cord blood gas). These data agree with our sample, where induced births presented a 1.828 times greater risk of obtaining a pathological fetal pH than did spontaneous births, yielding a statistically significant bivariate analysis between initiation of birth and fetal acidosis (*p*: 0.03).

According to induction reason, intrauterine growth restriction is one of the main risk factors for intrapartum fetal acidosis in our results and in those of Kehl et al. (35), Pinton et al. (36), and Massimiani et al. (37), with more postpartum transfers to the neonatal intensive care unit and high perinatal mortality and morbidity (35), this placental dysfunction predisposes the fetus to intrapartum fetal compromise.

The reasons for induction detected in the sample: pregnancy indications (prolonged pregnancy, premature rupture of membranes), maternal indications (maternal disease unrelated to pregnancy and pregnancy-induced hypertension), and fetal indications (retarded intrauterine growth, risk of loss of fetal well-being, and oligohydramnios) (38) show similarities with the literature where induction of labor was also associated with adverse neonatal outcome (17).

Uterine contractions in labor result in a 60% reduction in uteroplacental perfusion, causing transient fetal and placental hypoxia. A healthy fetus with a normally developed placenta is able to accommodate this transient hypoxia by activation of the peripheral chemoreflex, resulting in a reduction in oxygen consumption and a centralization of oxygenated blood to critical organs, namely, the heart, brain, and adrenals. Providing there is adequate time for placental and fetal reperfusion between contractions, these fetuses will be able to withstand prolonged periods of intermittent hipoxia and avoid severe hypoxic injury (39). In induction of labor, these contractions are produced artificially on a fetus in which the end of pregnancy has been indicated for different non-physiological reasons, thus increasing the risk of fetal acidosis due to deterioration in gas exchange. While decreasing the risk of shoulder dystocia or meconium aspiration syndrome, induction may be a risk of adverse consequences such as uterine hyperstimulation, fetal asphyxia, postpartum hemorrhage, or uterine rupture, being related to further intervention such as the use of epidural analgesia, instrumental delivery, or emergency cesarean section (40).

Studies (41–43) also show a strong association between instrumented delivery and fetal acidosis: There is a large volume of scientific literature that maintains that instrumented vaginal birth increases the incidence of fetal compromise compared with eutocic vaginal birth (44–47), a conclusion which bears similarity to our results. Kapaya et al. (46) compared the different types of delivery, finding that instrumented birth corresponded to the highest percentage of neonates with pathological pH values (45%) and elective cesarean the lowest (0.3), and Zaigham et al. (48), found a significantly lower pH, pCO_2_, pO_2_, and sO_2_ in mothers delivering vaginally in comparison with planned cesarean sections. The significant differences in acid–base values according to mode of delivery is based on processes such as during vaginal delivery, the sheer force of uterine contractions and bearing down results in impaired oxygenation and anaerobic glycolysis with lactate accumulation in both the mother and fetus (48).

Finally, an association between fetal acidosis and increased duration of dilation (49, 50), duration of expulsion (51, 52), and total duration of delivery (49, 51) has also been observed. Our study shows a significant decrease in umbilical artery pH mean values when the duration of dilatation, duration of expulsion, and total duration of delivery were higher than the mean and fetal acidosis (<7.20) when total duration of labor was above average. Altman et al. (53), in comparing expulsion durations of <1 h with expulsion durations of 1 to >2 h, associated the latter with an 80% higher risk of 5-min Apgar scores of <7, with the risks increasing gradually with longer durations, and also linked expulsion stages of >4 h with a two-time greater risk of an Apgar test score of <7. This risk increase is similar to that found in our sample, where the risk of acidosis was twice as high in the group of women with an expulsion time greater than the sample average. Yi et al. (17) relate a prolonged second phase of delivery to serious neonatal outcomes: 5-min Apgar test scores of <3, severe respiratory distress syndrome, severe acidosis, admission to neonatal intensive care unit, and fetal or neonatal death. The reason could be delayed labor, which might be causing the fetus to be involved in labor for a long time that carries a higher risk of birth trauma and asphyxia (54). These data are explained by the fact that the longer the duration of the labor process, that is, dilation, expulsion, and therefore the total duration, there is an association with fetal and maternal exhaustion and consequently in the production of fetal compromise resulting in acidosis at birth (55).

Prolonged labor was associated with a higher percentage of primiparous, higher gestational age, instrumental delivery, and induction of labor, variables that had an impact significant increase in the risk of poor outcomes (56–58).

There is a need for a greater body of scientific work on the concept of acidosis and its relationship with both clinical and sociodemographic variables, using neonatal cord blood gasometry - a measurement tool based on objective and internationally regarded analysis - in the diagnosis.

## Strengths and Limitations

The strengths of the study lie in that it investigates not only factors associated with acidosis at birth, as in previous studies, but also factors that influence the reduction of average umbilical artery pH values, using an objective test in the form of umbilical artery gasometry analysis at birth applied to a relatively large cohort of neonates selected at random. Other strengths were that blood sampling was conducted by trained professionals and all blood gas samples were analyzed immediately upon procurement, aided by a blood gas analyzer machine located within the Labor and Delivery department of the Hospital.

Among the limitations due to the type of study, it is difficult to include pathological variables of low incidence, the study could benefit from the use of a multicenter approach and observational studies.

Systematic error analysis was carried out on the following, information bias: the data collection was carried out with the exhaustive review by a single investigator of each patient's clinical record in computerized format (Selene), thus, controlling interobserver variability with uniform collection criteria; confounding bias: for the main outcome variables, the corresponding multivariate adjustment was made considering that these variables could act as confounders; selection bias: before starting data collection, the sample size was calculated based on the incidence of the study phenomenon.

## Conclusions

Several factors, such as gestational age, primiparity, labor induction, vaginal delivery, and above-average duration of dilation, expulsion, and total labor duration have a significant association with the decrease in the averages of umbilical artery pH at birth; however, only induction of labor, vaginal delivery, and total duration of labor are associated with acidosis (<7.20) of the same.

## Data Availability Statement

The raw data supporting the conclusions of this article will be made available by the authors, without undue reservation.

## Ethics Statement

The studies involving human participants were reviewed and approved by Comité de Investigación del Área III de Salud. (Hospital Rafael Méndez Lorca). Written informed consent for participation was not required for this study in accordance with the national legislation and the institutional requirements.

## Author Contributions

JH, MP, and PP: conceptualization, methodology, investigation, writing—original draft preparation, visualization, supervision, and project administration. JH and MP: software, validation, formal analysis, resources, data curation, and writing—review and editing. All authors contributed to the article and approved the submitted version.

## Conflict of Interest

The authors declare that the research was conducted in the absence of any commercial or financial relationships that could be construed as a potential conflict of interest.
